# Characterization of Hydrophobic Peptides in the Presence of Detergent by Photoionization Mass Spectrometry

**DOI:** 10.1371/journal.pone.0079033

**Published:** 2013-11-13

**Authors:** Aïcha Bagag, Jean-Michel Jault, Nazha Sidahmed-Adrar, Matthieu Réfrégiers, Alexandre Giuliani, François Le Naour

**Affiliations:** 1 Inserm, U785, Villejuif, France; 2 Université Paris-Sud 11, Institut André Lwoff, Villejuif, France; 3 Université Joseph Fourier-Grenoble 1, Institut de Biologie Structurale, Grenoble, France; 4 CNRS, UMR 5075, Grenoble, France; 5 CEA, Institut de Biologie Structurale, Grenoble, France; 6 Synchrotron SOLEIL, Gif-sur-Yvette, France; 7 INRA, UAR 1008 CEPIA, Nantes, France; Swiss Institute of Bioinformatics, Switzerland

## Abstract

The characterization of membrane proteins is still challenging. The major issue is the high hydrophobicity of membrane proteins that necessitates the use of detergents for their extraction and solubilization. The very poor compatibility of mass spectrometry with detergents remains a tremendous obstacle in studies of membrane proteins. Here, we investigated the potential of atmospheric pressure photoionization (APPI) for mass spectrometry study of membrane proteins. This work was focused on the tetraspanin CD9 and the multidrug transporter BmrA. A set of peptides from CD9, exhibiting a broad range of hydropathicity, was investigated using APPI as compared to electrospray ionization (ESI). Mass spectrometry experiments revealed that the most hydrophobic peptides were hardly ionized by ESI whereas all peptides, including the highly hydrophobic one that corresponds to the full sequence of the first transmembrane domain of CD9, were easily ionized by APPI. The native protein BmrA purified in the presence of the non-ionic detergent beta-D-dodecyl maltoside (DDM) was digested *in*-solution using trypsin. The resulting peptides were investigated by flow injection analysis of the mixture followed by mass spectrometry. Upon ESI, only detergent ions were detected and the ionic signals from the peptides were totally suppressed. In contrast, APPI allowed many peptides distributed along the sequence of the protein to be detected. Furthermore, the parent ion corresponding to the first transmembrane domain of the protein BmrA was detected under APPI conditions. Careful examination of the APPI mass spectrum revealed a-, b-, c- and y- fragment ions generated by *in*-source fragmentation. Those fragment ions allowed unambiguous structural characterization of the transmembrane domain. In conclusion, APPI–MS appears as a versatile method allowing the ionization and fragmentation of hydrophobic peptides in the presence of detergent.

## Introduction

Membrane proteins represent about one-third of the genome [1]. The interfacial location of these proteins between the outside and the inside of the cell is related to their key role in cellular functions such as cell adhesion, signal transduction, molecular transport, endocytosis and trafficking. Membrane proteins play also a major role in infectious diseases as receptors for viruses and parasites. Thus, the membrane proteome exhibits a high potential for characterizing new biological markers or targets for therapeutics [2], [3].

Despite its importance, the membrane proteome has not been explored in depth yet, owing to important challenges pertaining to the characterization of membrane proteins [3]–[6]. The major issue in studies on membrane proteins resides in their high hydrophobicity. As embedded in the lipid bilayer, their extraction and solubilization require the use of detergents [7], [8]. The intrinsic hydrophobicity of membrane proteins added with the presence of detergents in membrane preparations remain important limitations in further proteomic and mass spectrometry analysis. The main proteomic approach based on two-dimensional electrophoresis (2DE) is not suitable for membrane proteins separation because of the difficulties in extracting and solubilizing them in the isoelectric focusing (IEF) sample buffer. Membrane proteins, especially those with multiple transmembrane domains (TM), are poorly soluble in these mixtures [4]. In addition, membrane proteins exhibit generally alkaline isoelectric point (pI) that further limits their separation in 2DE [4] as they tend to precipitate at their pIs during IEF [5]. Hence, conventional 2DE is only able to resolve membrane proteins with no more than two TM domains whereas those with multiple TM domains could not be really identified by 2DE [3]–[5]. As a consequence, membrane proteins are under-represented in many proteome profiles. On the other hand, liquid chromatography is also not suitable for investigating membrane proteins or peptides preparations because the detergents, in particular non-ionic ones, can stick on the column thus yielding intense signal in the mass spectra along the LC–MS analysis. A tremendous blockage for the analysis of membrane proteins is the incompatibility of the presence of detergents with ionization methods mostly used in mass spectrometry, in particular electrospray ionization (ESI). Indeed, it is well known that detergents and surfactants lead to signal suppression from peptides and proteins [8]–[10]. Therefore, many complicated and time-consuming separation techniques were implemented in order to tentatively remove detergents before mass spectrometry analysis [11]–[14]. These methodologies are not versatile enough for investigating the membrane proteome.

The alternative proteomic approach combining protein separation on sodium dodecyl sulfate polyacrylamide gel electrophoresis (SDS–PAGE) followed by *in*-gel digestion and shotgun analysis (LC–MS/MS) has been shown to be more adapted than other approaches to membrane proteins and successful studies have been reported [15]–[19]. However, membrane proteins are identified essentially by their hydrophilic peptides resulting from the trypsin digestion. This is due to the inherent lack of trypsin cleavage sites (K or R) in the hydrophobic regions of membrane proteins and to the tendency of the hydrophobic peptides to aggregate and to precipitate in aqueous solutions after removal of detergent. In addition, the poor signal obtained with hydrophobic peptides using conventional soft ionization techniques such as ESI in mass spectrometry analysis contributes also to the difficulties in analysing such peptides. Thus, there is still under-representation of highly hydrophobic membrane proteins in shotgun proteomics [11].

Novel methods are needed for investigating the membrane proteome. A mass spectrometry technique adapted to the ionization of hydrophobic molecules and tolerant to detergents would solve the current blockage in studies of membrane proteins. Atmospheric pressure photoionization (APPI) has been developed in the beginning of the century [20], [21] and is thus the latest of the atmospheric pressure ionization techniques. APPI has been recently used in studies of macromolecules and has been reported as promising for investigating hydrophobic compounds [22]–[25]. However, no studies have been performed on membrane proteins.

In this report, we demonstrated that APPI–MS allows the ionization and fragmentation of highly hydrophobic peptides from membrane proteins despite the presence of an important amount of detergent. Thus, APPI mass spectrometry appears as a powerful approach for structural and proteomic studies on membrane proteins.

## Materials and Methods

### Chemical Reagents and Peptides

The solvent and reagents were high purity or HPLC grade. Methanol was purchased from Prolabo (Fontenay-sous-Bois, France). Water used was bidistilled and filtered on Millipore cartridges (18 MΩ). Toluene 99,5% (from SDS, Peypin, France) was used as dopant and introduced at various flow rates using a Harvard syringe pump model 22 (Harvard, Holliston, MA, USA). The detergent SDS was purchased from Sigma (Saint Quentin, France). The studied peptides come from the human CD9 protein sequence (Table 1). The peptide p12–36 (YLLFGFNFIFWLAGIAVLAIGLWLR) was synthesized at IFR 83 (Paris, France), and the other peptides were synthesized by Genepep (Montpellier, France). The peptides were dissolved in a mixture H_2_O/CH_3_OH (10∶90, v/v) at a concentration of 20 µmol.L^−1^.

**Table 1:** Peptides from the human CD9 membrane protein.

Peptide #	Position	Number AA	Sequence	Monoisotopicmass (M+H)^+^	GRAVY	Mixture #
1	120–131	12	EVQEFYKDTYNK	1563.73	−1.800	M1
2	104–131	28	**IAAAIWGY**SHKDEVIKEVQEFYKDTYNK	3345.68	−0.711	M2
3	1–11	11	MPVKGGTKCIK	11161.65	−0.155	M3
4	84–90	7	ESQCMLG	767.31	0.000	M4
5	104–119	16	**IAAAIWGY**SHKDEVIK	1800.96	0.106	M5
6	101–119	19	**AIEIAAAIWGY**SHKDEVIK	2114.13	0.237	M6
7	104–114	11	**IAAAIWGY**SHK	1216.64	0.355	M7
8	76–90	15	LGAAGAVQESQCMLG	1433.69	0.607	M8
9	12–36	25	Y**LLFGFNFIFWLAGIAVLAIGL**WLR	2913.66	1.792	M9

Bold amino acids are located in transmembrane domains of the protein according to SwissProt. The GRAVY (grand average of hydropathicity) value for a peptide is calculated as the sum of hydropathy values of all the amino acids divided by the number of residues in the sequence (Kyte and Doolittle, 1982).

### Membrane Preparation and Purification of BmrA

BmrA-enriched *E. coli* membranes were prepared as described previously [26] and kept frozen in liquid nitrogen. Purification of BmrA was performed at 4°C essentially as described previously [27]. Briefly, thawed membranes were diluted at 2 mg/ml proteins in buffer A (50 mM Tris/HCl, pH 8, 15% glycerol, 100 mM NaCl, 10 mM imidazole, 1 mM dithiothreitol, 1 tablet/50 mL of antiproteases (complete EDTA-free from Roche), and 1 mM phenylmethylsulfonyl fluoride) in the presence of 1% *n*-dodecyl-β-D-maltoside (DDM). Ultracentrifugation was then performed (185 000 g, 1 h), and supernatant was incubated 30 min in the presence of Ni^2+^-nitrilotriacetic acid-agarose. The slurry was then incubated overnight with 20 mL of buffer A in the presence of 0.05% DDM. The slurry was poured into a column and washed with 20 mL of buffer A containing 0.05% DDM. BmrA was eluted using a low volume (∼ 7 mL) of buffer A containing 250 mM imidazole in the presence of 0.05% DDM. Fractions containing the proteins were pooled and dialyzed twice against 1 liter of buffer containing 50 mM Tris/HCl, pH 8, 50 mM NaCl, 10% glycerol, 0.05% DDM, and 5 mM β-mercaptoethanol. Aliquotes were kept frozen in liquid nitrogen until use. Protein concentrations were estimated by using a “Coomassie Plus protein assay reagent” (Thermo Fisher Scientific, Courtaboeuf, France).

### *In*-solution Trypsin Digestion

The protein BmrA was digested in solution directly after purification and dialysis. BmrA at concentration of 10 µM in 200 µL containing 50 mM Tris/HCl, pH 8, 50 mM NaCl, 10% glycerol, 0.05% DDM, and 5 mM β-mercaptoethanol was reduced with 10 mM DTT (Sigma, Saint-Quentin Fallavier, France) at 56°C for 45 minutes followed by alkylation with 40 mM iodoacetamide (Sigma) at room temperature in the dark for 1 hour. The digestion was performed with trypsin (Sigma) in the ratio of trypsin to sample protein of 1/50 at 37°C overnight.

### Mass Spectrometry Analysis

Electrospray ionization (ESI) experiments were performed using the TurboIon source of a QStar Pulsar *i* (AB Sciex, Framingham, MA).

Photoionization experiments were carried out on the beamline DISCO [28] at synchrotron SOLEIL (Saint Aubin, France) using the Photospray™ source (AB Sciex), which has been modified to use synchrotron radiation as the photon source [24], [29]. The major advantages of synchrotron radiation as an UV source are high flux of photon and accordability in the 4 to 20 eV photon energy range, which allows using optimal photon energy for the ionization of the sample. The experiments were performed with toluene used as a dopant at the flow rate 10 µL.min^−1^ and the energy was set up at 9 eV. The thermospray mode has been achieved by switching off the VUV radiation that is performed by inserting an obturator valve on the optical path. All the other experimental parameters such as temperature, voltage or flow rate were kept identical to the APPI conditions.

The samples were injected by the flow-injection analysis (FIA) method, in which 20 µL of the sample solutions were loaded into an injection loop and next eluted with a mixture H_2_O/CH_3_OH (10∶90, v/v). The solvent was introduced into the photospray ionization source using a HPLC pump Agilent 1100 series (Agilent Technologies, Palo alto, CA, USA) at a flow rate of 200 µL.min^−1^. The nebulizer gas was dry air.

Mass spectra were recorded using a hybrid quadrupole-time-of-flight Qstar Pulsar *i* mass spectrometer (AB Sciex). Mass spectrometric instrumental parameters were adjusted in order to obtain the best signal-to-noise ratio and to minimize possible in source collision-induced dissociation (CID). Operating parameters for ESI experiments were: ISV (Ion Source Voltage) = 4000 V, DP_1_ (Declustering Potential 1) = 20 V, FP (Focusing Potential) = 100 V, DP_2_ (Declustering Potential 2) = 15 V. Operating parameters for APPI experiments were: Heating temperature = 500°C ISV (Ion Source Voltage) = 1200 V, DP_1_ (Declustering Potential 1) = 20 V, FP (Focusing Potential) = 100 V, DP_2_ (Declustering Potential 2) = 15 V. The gas flow which protects the lamp was fixed to 2 L.min^−1^. Source gas is pure nitrogen from a generator with less than 5 ppm of oxygen. Data were acquired and treated using the Analyst QS software (AB Sciex).

## Results and Discussion

### APPI allows the Ionization of Highly Hydrophobic Peptides

Atmospheric pressure photoionization (APPI) relies on the interaction of VUV photons with a heated pneumatic spray termed thermospray (TSP) that is generated in a similar way as for atmospheric pressure chemical ionization (APCI). The basic mechanism of APPI involves the direct photoionization of the analyte or APPI ion sources may be operated with adjunction of a molecule, called a dopant, which is easily photoionized [21], [30]. APPI can provide high ionization efficiency to both polar and non-polar compounds.

**Figure 1:**
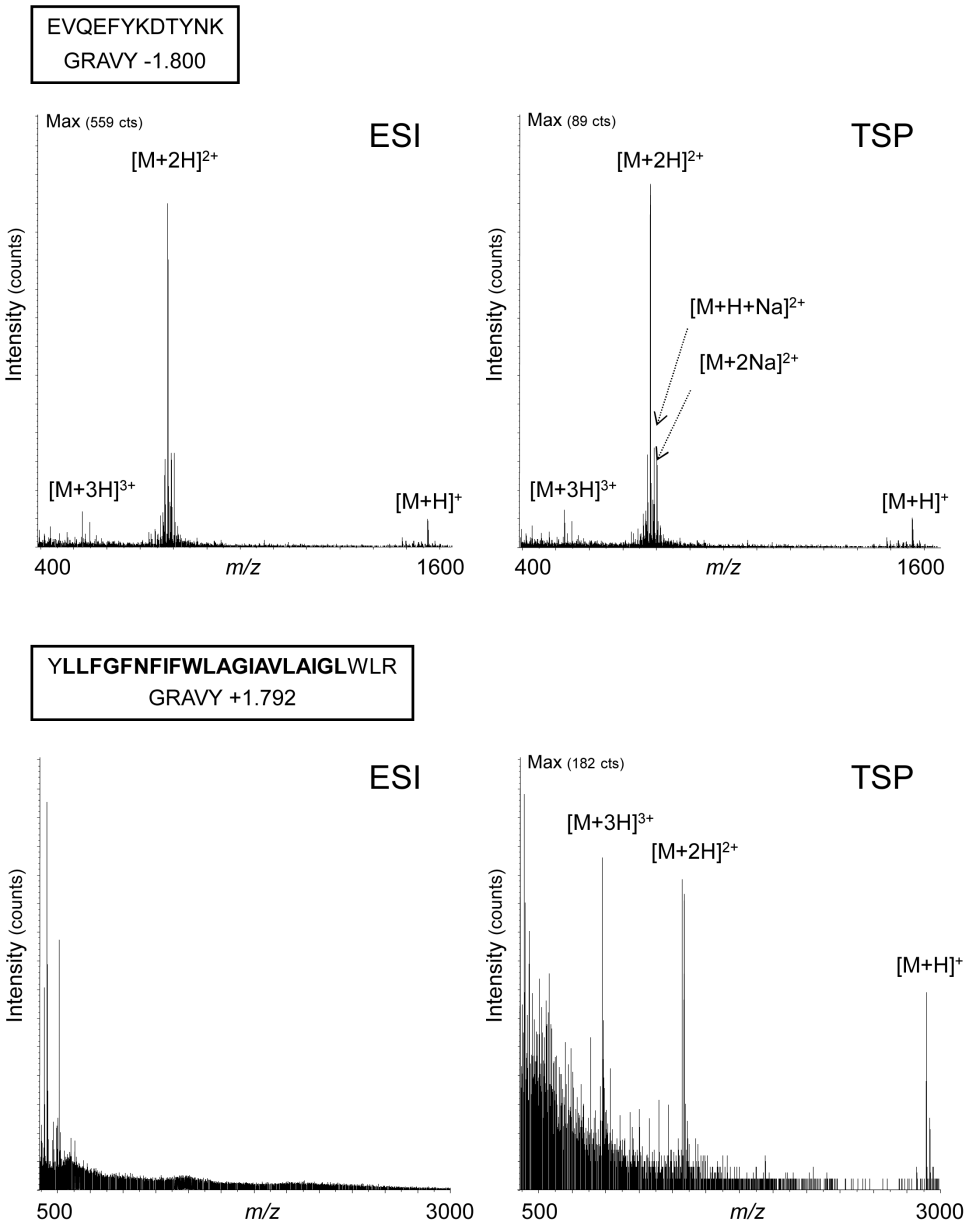
Ionization of two extreme hydrophilic and hydrophobic peptides in ESI or TSP. The hydrophilic peptide located in the second extracellular loop of the protein CD9 (aa 120–131) (upper panel) and the highly hydrophobic peptide corresponding to the first transmembrane domain (aa 12–36) (lower panel) were investigated by mass spectrometry using electrospray ionization (ESI) or thermospray (TSP) as ionization modes. The sequence corresponding to the transmembrane domain is in bold.

We have addressed the potential of APPI–MS for characterizing hydrophobic peptides focusing on the tetraspanin CD9. Tetraspanins that possess four transmembrane domains are major components of the biological membranes in which they organize membrane microdomains [16], [31]–[33]. A set of peptides from the tetraspanin CD9 exhibiting a grand average of hydropathicity index (GRAVY) ranking between −1.8 and +1.8 (Table 1) have been analysed under ESI and APPI conditions. A positive GRAVY index indicates a hydrophobic compound whereas a negative GRAVY index is obtained for hydrophilic compounds [34]. We first focused on the two peptides exhibiting the extreme Gravy indexes of −1.8 and +1.792, corresponding respectively to a hydrophilic tryptic peptide (aa 120–131) located in the second extracellular loop of the tetraspanin CD9 [35] and the full sequence of the first transmembrane domain (aa 12–36) of CD9. Mass spectrometry experiments were first performed using ESI and with the APPI source used as in thermospray (TSP) mode (ie without shining photons inside). Ionic signal was obtained with the hydrophilic peptide (aa 120–131) in both ionization processes ESI and TSP. In contrast, no signal was measured with the transmembrane domain in ESI whereas the peptide was ionized in TSP. The spectrum led to detect the mono-, di- and tri-charged forms of this large hydrophobic peptide (Fig. 1). Furthermore, mass spectrometry analysis on the other seven peptides individually demonstrated that the most hydrophobic peptides exhibiting a positive GRAVY index higher than +1 were not ionized in ESI whereas signals were always obtained with TSP (data not shown).

**Figure 2:**
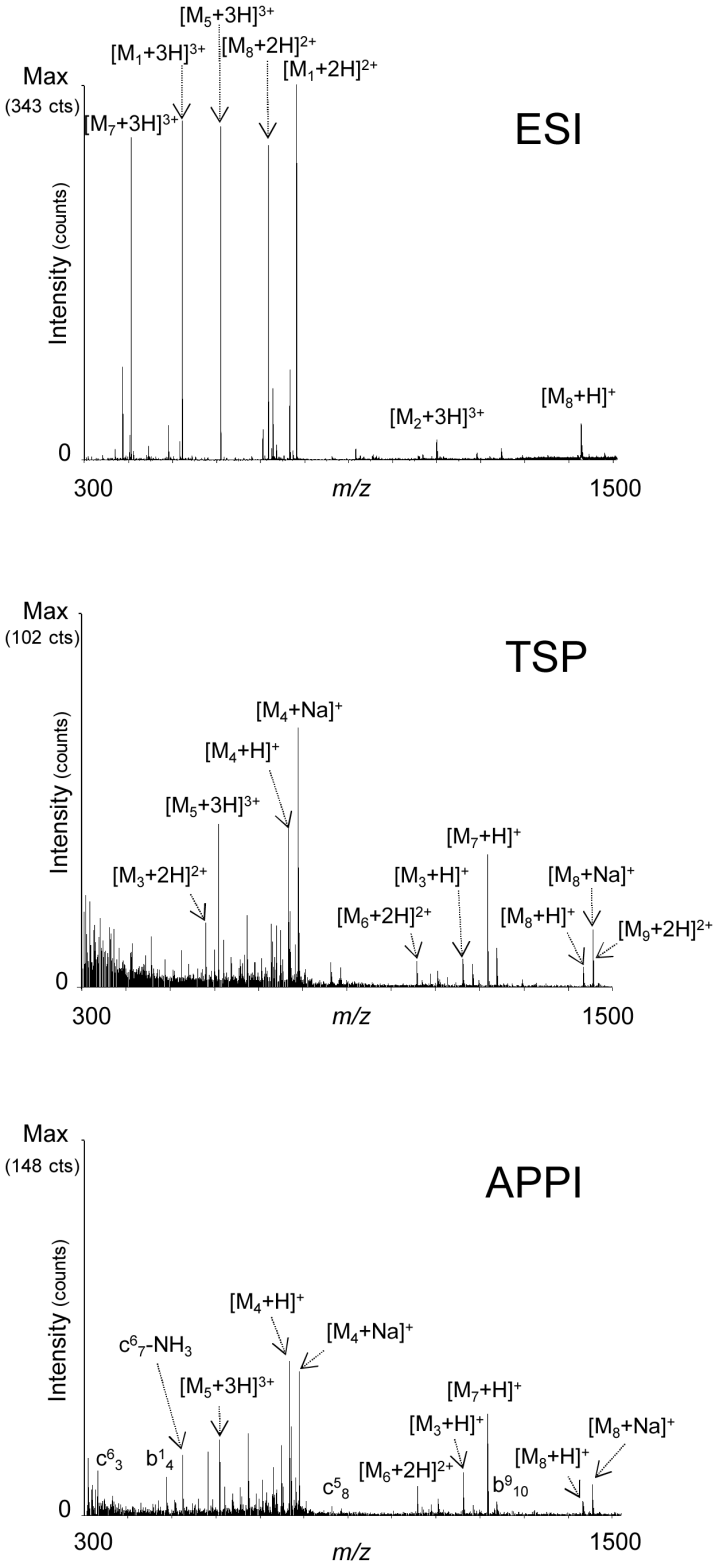
Ionization of a mixture of peptides from CD9 in ESI, TSP or APPI. Nine CD9 peptides exhibiting a broad range of hydropathicity were mixed at equimolar concentration. These peptides noted M1–M9 are described in the Table 1. The mixture was investigated by electrospray ionization (ESI) or by thermospray (TSP). Photoionization (APPI) was performed on the thermospray under dopant assisted conditions using toluene and 9 eV photons. For b- or c-ions, the peptide number is indicated as an exponent and fragment as an index.

**Figure 3:**
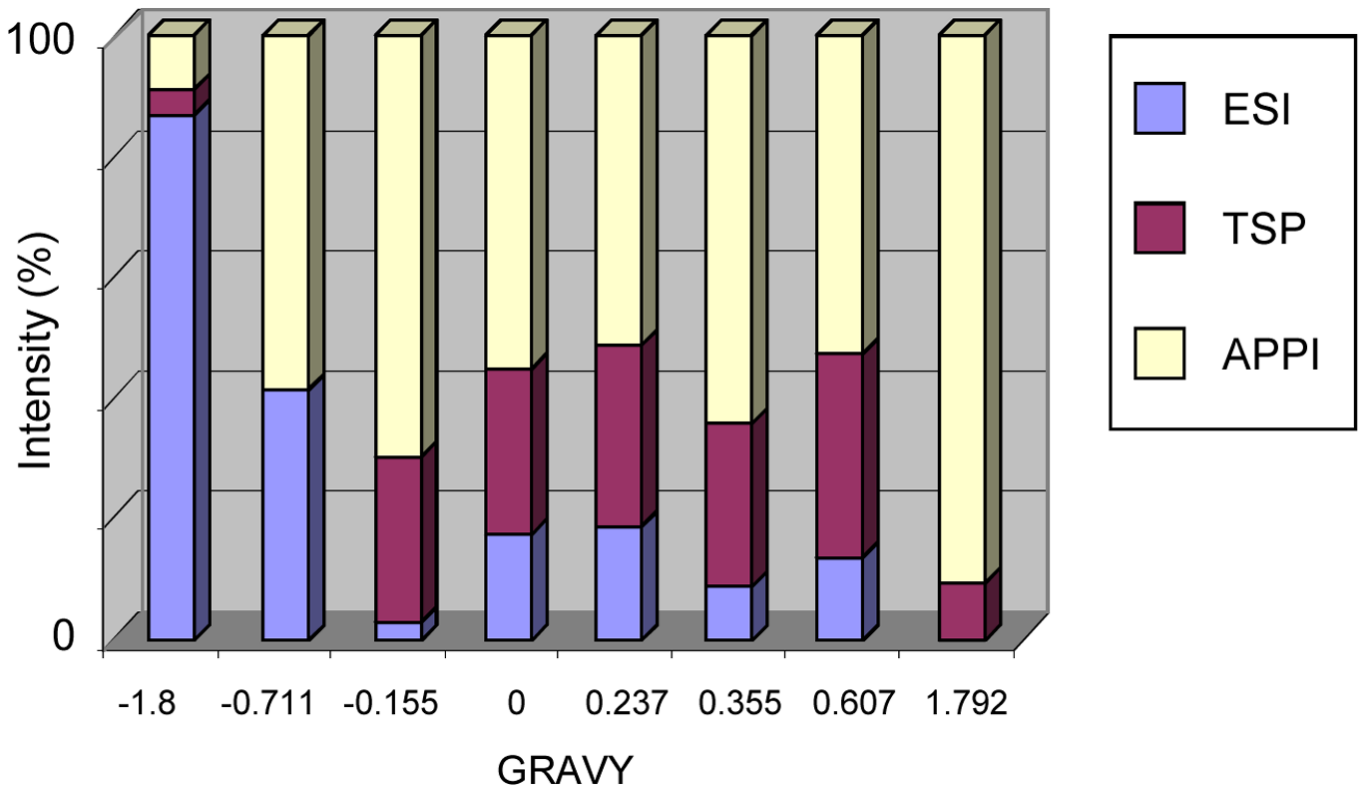
Ionization of peptides related to their hydropathicity. The mixture of peptides from CD9 was investigated in ESI, TSP or APPI. For each peptide, the intensity of the precursor plus the fragments ions obtained using the different ionization technique ESI, TSP or APPI were plotted in the diagram.

Investigations were further performed on a mixture of the nine peptides from CD9 (Table 1) with various degree of hydrophobicity (Fig. 2). After ESI–MS analysis, only five peptides were detected mostly in their di- and tri-charged forms and all the species were protonated. The most abundant peak ion in this spectrum corresponded to the most hydrophilic peptide (M1, aa 120–131) possessing a GRAVY index of −1.8. Moreover, we also detect some hydrophilic peptides such as M2 (aa 104–131, Gravy −0.711) and M5 (aa 104–119, Gravy +0.106) and two more hydrophobic M7 (aa 104–114, Gravy +0.355) and M8 (aa 76–90, Gravy +0.607) with similar intensities. It is interesting to note that the most hydrophobic peptide (M9, aa 12–36) was not observed under such conditions. With the same mixture of peptides, experiments were performed with the photospray source without photons as a thermospray (TSP) source (Fig. 2). In contrast to ESI–MS, the most hydrophilic peptides (M1 and M2) were no longer detected in those conditions but all hydrophobic peptides were observed in the mass spectrum including the most hydrophobic one (M9) with a GRAVY index +1.792. It should be noted that along with protonated species, we also observe some cationized species especially for M4 and M8. The cationized and protonated species observed under such conditions are transferred into the gas phase by a thermospray-like process, which is the desolvatation of charged species preformed in solution, as shown in previous works [36], [37]. Further mass spectrometry analyses were performed on the same mixture in APPI under dopant-assisted conditions using toluene and 9 eV photon energy (Fig. 2). Under these conditions, important *in*-source fragmentations were observed yielded mostly b- and c- ions as previously described [22]–[24], [37]. Interestingly, we remark that peptides with higher GRAVY index lead to more abundant fragmentations and thus lower detection of the precursor ions. For example, the precursor ion of the most hydrophobic peptide of the mixture (M9, Gravy +1.792) was not detected but we observed one fragment coming from the backbone cleavage leading to the b_10_ sequence ion. Deeper investigations have been carried out in order to decipher a behavior between the ionization efficiency versus the hydrophobicity of the studied peptides. We represented the intensity of the precursor and fragments ions for each ionization technique (ESI, TSP, APPI) in relation to the GRAVY index of the peptides in the mixture (Fig. 3). In ESI, we observed the best efficiency of ionization for peptide M1 which is explained by the negative value of its GRAVY index. The more the value of this parameter increases, the less the peptides are easily ionized in ESI. As a rule, all the peptides were easily ionized by photoionization. It is worth noting that APPI is more sensitive for hydrophobic compounds (Fig. 3). Altogether, these observations demonstrate that APPI is a powerful ionization technique for investigating hydrophobic peptides from membrane proteins.

### APPI allows Structural Investigation on Hydrophobic Peptides

**Figure 4:**
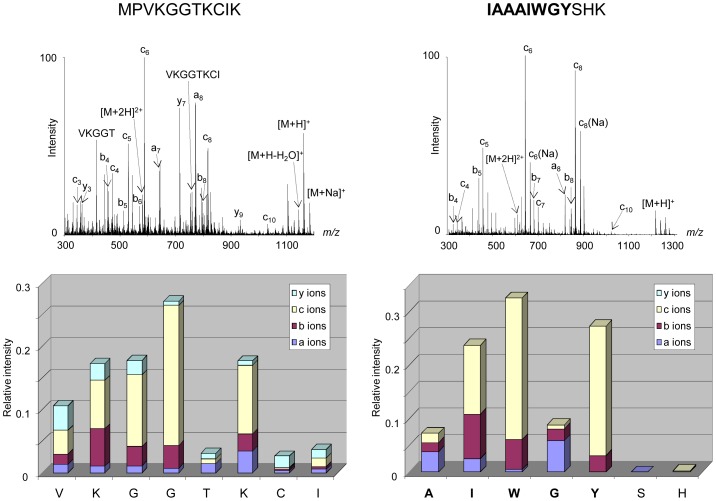
*In*-source fragmentation of peptides under APPI. Two peptides originating from the protein CD9 corresponding to a hydrophilic peptide (aa 1–11, Gravy −0.155) (left) and a hydrophobic peptide (aa 104–114, Gravy +0.355) (right) were investigated by APPI under dopant assisted conditions using toluene and 9 eV photons. The sequence embedded in the membrane is represented in bold. Along with precursor ions, abundant and intense fragment ions were detected in the mass spectra corresponding to *in*-source fragmentation of studied peptides. The intensities of each fragment species a-, b-, c- and y-ions were plotted in relation to the peptide sequence.

**Figure 5:**
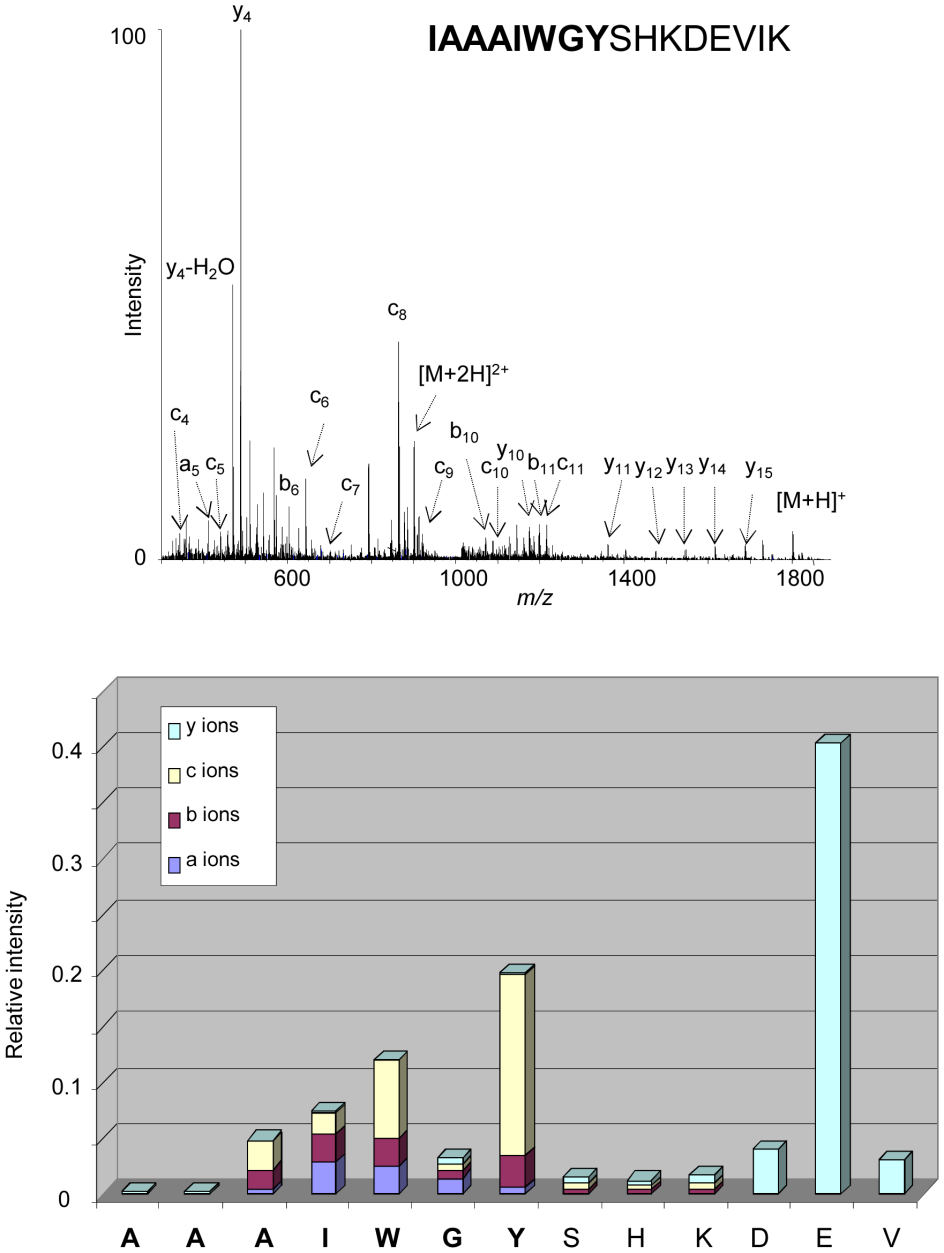
*In*-source fragmentation of a juxtamembrane peptide under APPI. The peptide (aa 104–119) corresponding to the C-terminal extremity of the third transmembrane domain and the N-terminal portion of the second extracellular loop of the tetraspanin CD9, was investigated by APPI under dopant assisted conditions using toluene and 9 eV photons. The portion of the peptide corresponding to the transmembrane domain is represented in bold. Abundant and intense fragment ions were detected in the mass spectrum corresponding to in-source fragmentation of the peptide. The intensities of each fragment species a-, b-, c- and y-ions were plotted in relation to the peptide sequence.

A major interest of APPI is the *in*-source fragmentation of peptides thus leading to structural studies. Fragmentations of peptides under APPI conditions were assigned partly to electron attachment to multiply protonated precursors [22]–[24], [37] and to reactions involving H radicals [38]. The fragmentations generated in source for one hydrophilic peptide (aa 1–11; Gravy −0.155) have been compared to those of a hydrophobic one (aa 104–114; Gravy +.0355) (Fig. 4). The fragment ions observed may be categorized into three groups: b−/y-, a- and c-, according to the Biemann nomenclature [39]. The peptides produced a-, b-, c- and y- ions under APPI conditions, independent from their GRAVY index (Fig. 4), thus demonstrating that the fragmentation process in APPI occurs also in the transmembrane domain where the amino acids are extremely hydrophobic. The study was further focused on the peptide positioned at a juxtamembrane region of the tetraspanin CD9 (aa 104–119; Gravy +0.106). The sequence of this peptide corresponds to the C-terminal extremity of the third transmembrane domain and the N-terminal portion of the second extracellular loop of the protein. The mass spectrum displays a weak signal corresponding to the protonated molecules along with numerous intense fragment ions. The ion abundances of each fragment ion (a-, b-, c- and y-) have been plotted as a function of the position of the amino acids in the peptides (Fig. 5). It appears that mainly c- sequence ions were produced in the hydrophobic portion of the peptide with also a minor contribution to a- and b- ions, whereas abundant y- ions were observed in the hydrophilic extremity of the peptide. This behaviour is different than that observed under CID activation [40], for which structural information are obtained mostly in the hydrophilic parts. Moreover, the nature of the sequence ions formed in the APPI source seems to correlate with the hydrophobic character along the peptidic backbone. These observations suggest that selective fragment ions may be generated under APPI indicative of the hydrophobicity of the peptide sequence.

### APPI-MS on the Membrane Protein BmrA in the Presence of Detergent

**Figure 6:**
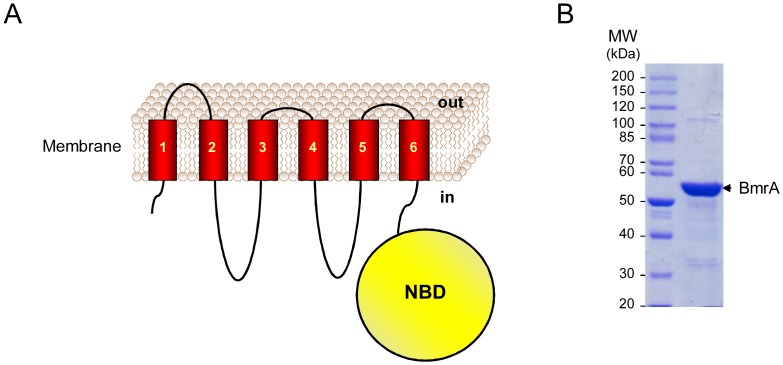
Topology and purification of the membrane protein BmrA. (A) The topology of the protein BmrA that exhibits six transmembrane domains and a nucleotide binding domain (NBD) is represented. (B) The purified protein was loaded on a gel and stained using Coomassie blue.

**Figure 7:**
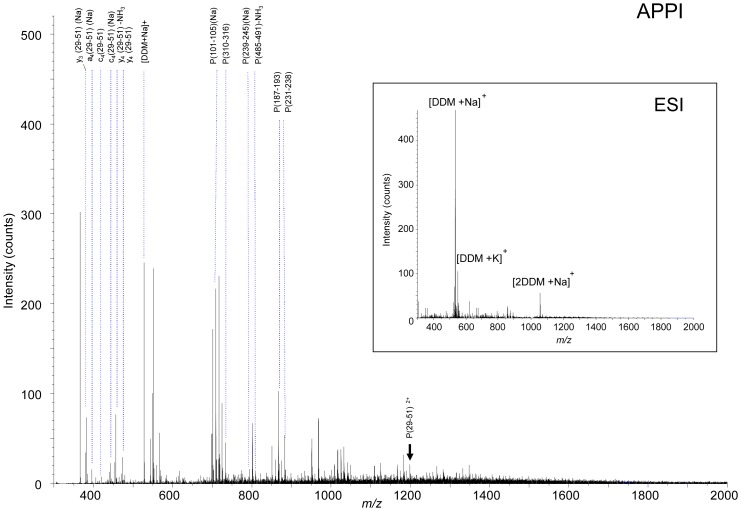
Mass spectrometry analysis after *in*-solution digestion of the protein BmrA. The purified protein BmrA (10 µM) was digested using trypsin in the solution containing 0.05% DDM, 50 mM NaCl, 50 mM Tris/HCl pH 8, 10% glycerol and 5 mM beta-mercaptoethanol. The mixture was investigated by mass spectrometry in ESI or APPI under dopant assisted conditions using toluene and 9 eV photons. The precursor ion *m/z* 1198.75 corresponding to the first transmembrane domain is labelled with a black arrow.

The presence of detergent is a major issue in mass spectrometry analysis because it leads to signal suppression of peptides and proteins [10]. The potential of photoionization for mass spectrometry analysis in the presence of detergent was addressed and compared with ESI. The study was focused on the multidrug BmrA protein that belongs to the ABC (ATP-binding cassette) transporters. These transporters are broadly expressed from bacteria to human. They are involved in import or export of a wide variety of substrates including amino acids, ions, sugars, complex organic molecules, peptides and even large proteins [41]. The structure of the protein BmrA has been characterized as an integral membrane protein exhibiting six putative transmembrane domains followed by one hydrophilic nucleotide-binding domain (NBD) [42] (Fig. 6A). We have performed mass spectrometry analysis on the purified BmrA protein (Fig. 6B) under the same conditions than those used for structural studies [26], [27], [43]–[45]. Thus, the protein was solubilized in 0.05% of the non ionic detergent beta-D-dodecyl maltoside (DDM) in the presence of salts (50 mM Tris/HCl and 50 mM NaCl). *In*-solution trypsin digestion of the protein was performed directly on such a mixture containing high concentration of salts and detergent. The mixture containing the resulting peptides of the enzymatic digestion was further analyzed in infusion on the mass spectrometer without any additional sample preparation. In electrospray ionization, we observed as expected total suppression of the ionic signal from any peptides and only peaks from the detergent according with our previous report [10]. Indeed, the ESI mass spectrum displays two very intense ion peaks at *m/z* 533 and 1043 corresponding to cationized monomer and dimer species of DDM (Fig. 7). In contrast, APPI–MS on the same mixture under dopant assisted conditions using toluene and 9 eV photon energy allowed detecting numerous intense peaks corresponding to peptides whereas the signal from DDM was negligible (Fig. 7). The behaviour of APPI may arise from processes taking place specifically in the APPI source leading to prevent sequestration of the peptide in detergent miscelles. Indeed, embedment of peptides or proteins inside detergent micelles has been proposed to be partly responsible for ion suppression but collision activation of such micelles lead to free peptides and proteins from detergent [10], [46], [47]. It is worth noting that the most intense ions in the *m/z* 300–2000 spectral domain of the mass spectrum were identified as parent as well as fragment ions generated from peptides of the protein BmrA (Table 2). These peptides were assigned to the BmrA sequence leading to approximately 25% of sequence coverage (Table 2). Interestingly, along with hydrophilic peptides, the ion *m/z* 1198.75 was observed that corresponds to the first transmembrane domain of the protein (aa 29–51, LAFALALSVVTTLVSLLIPLLTK). Careful scrutiny of the mass spectrum in the region between *m/z* 360 and 600 allowed observing fragment ions formed upon dissociation of this transmembrane domain (Table 2 and Fig. 7). The presence of intense sodium species was due to the high concentration of salt in the solution (NaCl, 50 mM). Finally, the series of sequence-specific fragment ions was covering the major part of the peptide sequence demonstrating that structural investigation of transmembrane domains can be carried out by atmospheric pressure photoionization on membrane proteins. Furthermore, APPI allows tandem experiments by CID. Indeed, the selection of the precursor ion (M+2H)^2+^ at *m/z* 1198.75 was possible even in the presence of an excess of DDM. The MS/MS spectrum of the *m/z* 1198.75 precursor ion lead to observe b- and y- ions related to sequence of the first transmembrane domain of BmrA. Although the fragment ions produced in CID where not as abundant as those generated by in-source fragmentation, they provide complementary information on the peptide sequence (Fig. S1).

**Table 2:** Peptides and fragments from digested BmrA in DDM detected using APPI-MS.

Fragments	*m/z* theoretical	*m/z* observed
c_3_(29–51)-NH_3_	332.1969	332.1889
y_3_(29–51)(Na)	383.2265	383.2102
a_4_(29–51)(Na)	397.2211	397.2655
c_4_(29–51)	420.2605	420.3518
c_4_(29–51)(Na)	442.2425	442.0157
y_4_(29–51) –NH_3_	457.3021	457.2807
y_4_(29–51)	474.3286	474.3096
P(487–491)	543.3613	543.3483
y_5_(29–51) –H_2_O	553.3708	553.2071
a_6_(29–51)	559.3602	559.2968
b_6_(29–51)	587.3552	587.2941
P(381–385)	609.3606	609.1533
b_7_(29–51)	700.4392	700.3894
P(101–105)(Na)	710.3848	710.3810
c_7_(29–51)	717.4658	717.3793
P(310–316)	733.3839	733.2586
P(239–245)(Na)	794.4496	794.4423
y_7_(29–51)(Na) –NH_3_	802.5050	802.4597
P(485–491) –NH_3_	810.4944	810.4294
a_9_(29–51)	858.5448	858.3659
P(187–193)	868.4709	868.2987
P(231–238)	882.4754	882.3804
P(476–484)	918.4826	918.4583
P(21–28)	922.4741	922.3220
P(580–587)	934.4299	934.4335
c_10_(29–51)	1002.6346	1002.6315
P(127–135)	1006.5238	1006.4464
P(232–241)-H_2_O–NH_3_	1032.5548	1032.4803
c_11_(29–51)	1103.6823	1103.5675
b_11_(Na)	1108.6378	1108.5974
y_11_(29–51)-H_2_O–NH_3_	1174.7811	1174.7658
a_12_(29–51)(Na)	1181.6905	1181.6616
P(29–51)	1198.7534	1198.7209
P(12–21)	1246.7783	1246.7231
b_13_(29–51)-H_2_O	1282.7769	1282.7308
c_13_(29–51)	1317.8140	1317.7903
P(231–242)	1307.7504	1307.6557
P(95–105)	1327.8096	1327.7714
y_12_(29–51)(Na)	1332.8478	1332.6574
P(580–592)	1404.6788	1404.7532
P(333–345)-NH_3_	1529.8071	1529.6605

The native protein BmrA was purified in 0.05% of the non ionic detergent beta-D-dodecyl maltoside (DDM) in the presence of salts (50 mM Tris/HCl and 50 mM NaCl). *In*-solution trypsin digestion was performed and the mixture was analyzed by in infusion on APPI–MS without any additional sample preparation. Ions obtained from the peptides (P) or fragments (a-, b-, c- or y-) are reported with the position along the protein sequence.

## Conclusion

This study positions APPI as a new approach for investigating membrane proteins. The high analytical potential of APPI was strengthened by ionization of extremely hydrophobic peptides such as transmembrane domains of membrane proteins, which are hardly ionized by electrospray ionization. Furthermore, we demonstrated the possibility to perform mass spectrometry analysis in the presence of an excess of detergent, in particular the non ionic detergent DDM. Non ionic detergents are usually employed for their ability to break lipid-lipid or lipid-protein interactions and to isolate native protein complexes because there are relatively mild compared to ionic detergents. The incompatibility of the presence of those detergents with the conventional ionization technique ESI mostly used in proteomics constitutes a tremendous bottleneck that still blocks the mass spectrometry analysis of membrane proteins. In contrast, APPI allows mass spectrometry analysis without any additional sample preparation. *In*-source fragmentation generated upon APPI allows yielding abundant fragment ions, which provide structural information on the studied peptides. Indeed, the selective fragmentation of juxtamembrane peptide leading c- ions in the transmembrane portion (hydrophobic), and y- ions in the extracellular portion (hydrophilic) is very appealing to locate the limits of the transmembrane domain. It should be noted that our results, though obtained using the coupling of an APPI source to a synchrotron radiation beamline, are clearer transposable to any laboratory APPI source using a krypton discharge lamp. On the other hand, the combination of photoionization mass spectrometry with liquid chromatography may open a large field of applications in proteomics. In conclusion, photoionization mass spectrometry appears as a versatile method allowing ionization and fragmentation of hydrophobic peptides in the presence of detergent. Therefore, we anticipate that photoionization mass spectrometry will open a new avenue in structural and proteomic studies on membrane proteins.

## Supporting Information

Figure S1 **Tandem mass spectrometry analysis of the first transmembrane domain of BmrA under APPI conditions.** After in-solution digestion of the protein BmrA and mass spectrometry analysis under APPI conditions, the precursor ion m/z 1198.75 was selected and fragmented by CID. The resulting MS/MS spectrum is shown.
(TIF)
