# Supplementary figures and images for: Characterization of Hydrophobic Peptides in the Presence of Detergent by Photoionization Mass Spectrometry
Source: PLoS One. 2013 Nov 13;8(11):e79033. doi: 10.1371/journal.pone.0079033 (PMC3827311; doi:10.1371/journal.pone.0079033)

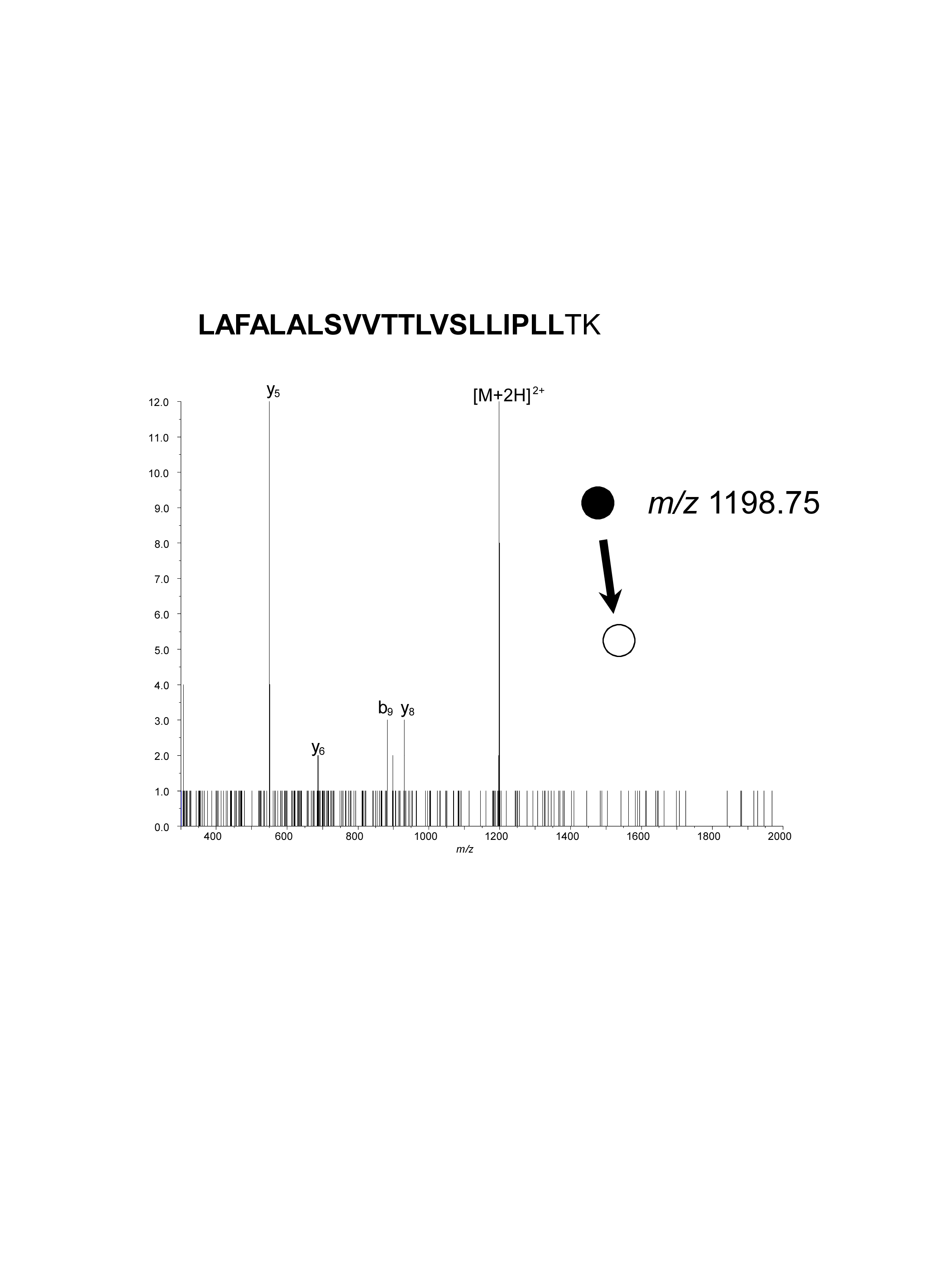

Supplement: Figure S1 — Tandem mass spectrometry analysis of the first transmembrane domain of BmrA under APPI conditions. After in-solution digestion of the protein BmrA and mass spectrometry analysis under APPI conditions, the precursor ion m/z 1198.75 was selected and fragmented by CID. The resulting MS/MS spectrum is shown. (TIF) [file pone.0079033.s001.tif]
